# CT radiomics machine learning for predicting treatment response in unresectable stage III-IV NSCLC: insights from dual chemo-immunotherapy and immunotherapy cohorts

**DOI:** 10.1007/s11547-026-02270-5

**Published:** 2026-08-10

**Authors:** Silvia Gigli, Lucia Malzone, Nicholas Landini, Roberta Valerieva Ninkova, Sandrine Riccardi, Giacomo Bonito, Laura Pappalardo, Alain Gelibter, Lucia Manganaro, Paolo Ricci

**Affiliations:** 1 Department of Diagnostic Imaging, Sandro Pertini Hospital, Via Dei Monti Tiburtini 385, 00157 Rome, Italy; 2 Department of Radiological, Oncological and Pathological Sciences, Policlinico Umberto I Hospital, Sapienza University of Rome, Viale Regina Elena 324, 00161 Rome, Italy; 3 Department of Emergency Radiology, Policlinico Umberto I Hospital, Sapienza University of Rome, Viale del Policlinico 155, 00161 Rome, Italy

**Keywords:** Radiomics, Machine learning, Advanced-stage unresectable NSCLC, Chemo-immunotherapy, Immunotherapy, CT, Predictive biomarkers

## Abstract

**Purpose:**

The purpose of this study was to develop a machine learning model based on radiomic features extracted from baseline contrast-enhanced CT scans for predicting early treatment responses in two cohorts of patients with stage III-IV NSCLC treated with distinct therapeutic regimens (first-line chemo-immunotherapy or immunotherapy alone).

**Methods:**

In this retrospective bicentric study including two cohorts, patients with a confirmed diagnosis of advanced NSCLC (stage III-IV) were retrospectively collected. At Center 1, after application of the exclusion criteria, 90 eligible patients (52 treated with CHT/IT and 38 with IT) were included for model development and internal validation. An independent external cohort from Center 2 comprised 40 additional patients, including 20 treated with CHT/IT and 20 with IT, and was used for external validation. Radiomic features were extracted from segmented tumor volumes using the Trace4Research™ platform, and multiple machine learning models, including random forest, support vector machine, K-nearest neighbors, multilayer perceptron, and logistic regression, were trained and compared.

**Results:**

In the CHT/IT cohort, seven radiomic features were retained after outlier removal. Among the tested models, the support vector machine (SVM) classifier showed the best performance, with a ROC-AUC of 0.90 (95% CI: 0.82–0.97), accuracy of 82%, sensitivity of 82%, specificity of 83%, positive predictive value of 67%, and negative predictive value of 94% (*p* < 0.005). In the IT cohort, 29 radiomic features were retained, and the SVM again achieved the best performance, with a ROC-AUC of 0.84 (95% CI: 0.73–0.95), accuracy of 77%, sensitivity of 82%, specificity of 70%, positive predictive value of 80%, and negative predictive value of 83% (*p* < 0.05). External validation supported the robustness and generalizability of both models.

**Conclusions:**

Machine learning models based on CT-derived radiomic features may enable accurate and noninvasive early prediction of treatment response in patients with advanced NSCLC receiving chemo-immunotherapy or immunotherapy alone, supporting the potential role of radiomics as an imaging biomarker for treatment stratification and personalized therapeutic planning.

**Supplementary Information:**

The online version contains supplementary material available at https://doi.org/10.1007/s11547-026-02270-5.

## Introduction

Non-small cell lung cancer (NSCLC) accounts for more than 85% of all lung cancers and remains one of the leading causes of cancer-related death worldwide. Because it is often diagnosed at an advanced stage, prognosis is frequently poor and therapeutic options may be limited [1, 2].

In patients with advanced unresectable NSCLC without targetable mutations, first-line treatment is mainly guided by PD-L1 (Programmed Death-Ligand 1) expression [3, 4]. Patients with a PD-L1 tumor proportion score (TPS) ≥ 50% are generally treated with single-agent immunotherapy, whereas those with PD-L1 < 50% usually receive chemotherapy and immunotherapy. However, treatment choice is also influenced by several clinical factors, including histology, disease burden, performance status, and comorbidities. The introduction of PD-1/PD-L1 inhibitors has significantly improved overall survival (OS) and progression-free survival (PFS), as shown in multiple phase III trials [5, 6].

Despite these advances, early assessment of treatment response remains challenging and is crucial for optimizing therapeutic strategies and improving patient management [7]. Contrast-enhanced computed tomography (CT) is the standard imaging modality for staging and response evaluation [8]. However, conventional CT assessment is still largely based on morphological criteria and may not fully capture subtle imaging characteristics associated with treatment outcome [9].

Radiomics has emerged as a promising approach for the quantitative characterization of tumors through the extraction of high-dimensional features from routine medical images. By converting image information into quantitative data, radiomics may provide a noninvasive means of describing tumor heterogeneity and microenvironmental characteristics. Several studies have reported the prognostic and predictive value of radiomic features in different malignancies, including NSCLC, suggesting a potential role for radiomics as an imaging biomarker for treatment response and survival prediction [10–12].

This approach may be particularly relevant in advanced NSCLC, where different systemic treatments are likely to produce distinct biological effects within the tumor. Cytotoxic chemotherapy mainly acts on rapidly proliferating tumor cells and may induce necrosis and more homogeneous tissue changes. In contrast, immunotherapy acts through modulation of the tumor–immune microenvironment and may be associated with more heterogeneous patterns related to immune infiltration, stromal remodeling, and inflammatory response [12]. These treatment-related biological differences may be reflected by distinct radiomic signatures on baseline contrast-enhanced CT images, supporting the development of treatment-specific predictive models.

Because radiomic data are high-dimensional and complex, advanced analytical methods are needed to translate these features into clinically useful information [13]. In this setting, machine learning (ML) offers a valuable tool for identifying patterns associated with treatment response and outcome [14]. By integrating radiomic features into ML models, it may be possible to improve early prediction of treatment response in patients with advanced NSCLC [15–17].

The aim of this study is to develop and externally validate machine learning models based on radiomic features extracted from baseline contrast-enhanced CT scans for the early prediction of treatment response in patients with unresectable stage III-IV NSCLC treated with first-line chemo-immunotherapy or immunotherapy alone.

## Methods

### Cohort selection

This retrospective bicentric study included two cohorts of patients with a confirmed diagnosis of advanced non-small cell lung cancer (stage III or IV) identified between December 2023 and April 2025 at two independent institutions in Rome, Italy (Center 1: Policlinico Umberto I Hospital; Center 2: Sandro Pertini Hospital). Written informed consent for the use of clinical data was obtained from all patients. The study was conducted in accordance with the ethical standards of the institutional and research committees of both centers, as well as with the 2013 Declaration of Helsinki and its subsequent amendments. Institutional Review Board approval was obtained under protocol number 0473/2024.

The inclusion criteria were: age ≥ 18 years; histopathological confirmation of NSCLC; availability of a baseline CT scan performed within 30 days before the start of systemic therapy and an early reassessment CT scan obtained 12–16 weeks after treatment initiation; receipt of first-line immunotherapy or chemo-immunotherapy; and the presence of at least one measurable target lesion (≥ 10 mm in the longest diameter) on baseline CT imaging.

The exclusion criteria were: absence of baseline or follow-up CT scans; incomplete clinical or radiological data; difficulty in lesion delineation on CT images, for example when the lesion was indistinguishable from atelectasis; inadequate CT image quality due to metal or motion artifacts; renal impairment precluding contrast medium administration (glomerular filtration rate < 30 mL/min); treatment with targeted therapy for actionable molecular alterations or radiotherapy; concurrent malignancies; and a history of prior thoracic surgery.

**Fig. 1:**
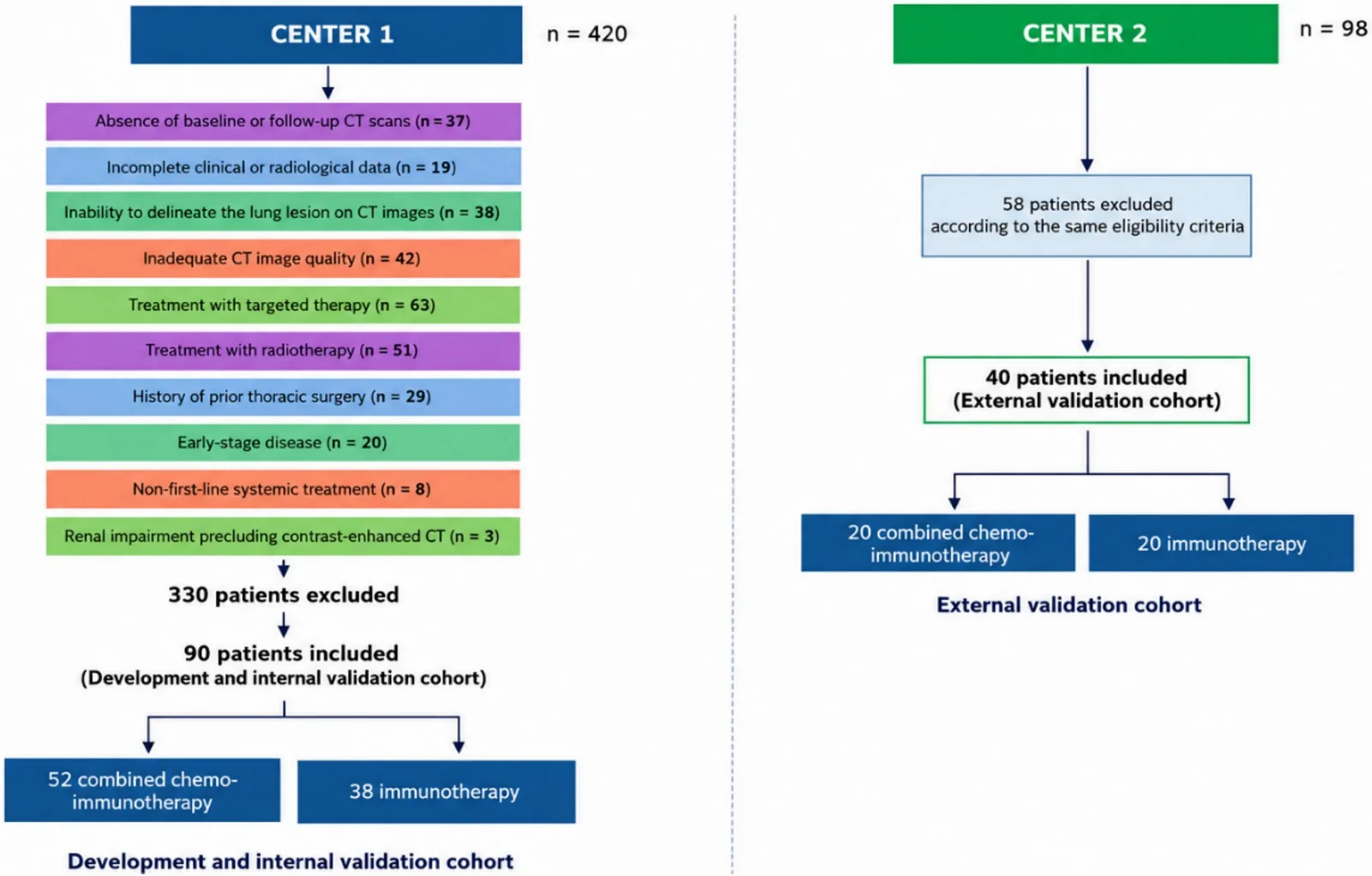
Flow diagram of patient selection and cohort allocation. Center 1 constituted the development and internal validation cohort, including 90 patients (52 treated with chemo-immunotherapy and 38 with immunotherapy). Center 2 constituted the independent external validation cohort, including 40 patients (20 treated with chemo-immunotherapy and 20 with immunotherapy). CT, computed tomography

At Center 1, 420 patients were initially screened. After application of the exclusion criteria, 90 patients were included in the internal cohort, including 52 treated with chemo-immunotherapy and 38 treated with immunotherapy alone. At Center 2, 98 patients were screened and 58 were excluded, yielding an external validation cohort of 40 evaluable patients, with 20 patients in each treatment group. The patient selection process is shown in Fig. 1.

### Data collection

To enable standardized data collection and analysis among participating centers, a dedicated clinical database was developed. All procedures for data collection and management were carried out in line with established protocols to safeguard privacy and ensure data security. Clinical and demographic data were retrospectively collected encompassing the following variables: age, sex, smoking status, tumor histology, PD-L1 expression, baseline carcinoembryonic antigen (CEA) and lactate dehydrogenase (LDH) levels, comorbidities, and therapy-related toxicities. Tumor staging (cT, cN, cM) was reassessed according to the 9th edition of the TNM classification for lung cancer [18]. Performance status was assessed using the Eastern Cooperative Oncology Group (ECOG) scale [19]. PD-L1 expression was measured through immunohistochemistry and reported as the percentage of tumor cells exhibiting membranous staining.

### Treatment protocols

First-line systemic treatment was selected according to PD-L1 expression and tumor histology. Patients with PD-L1 expression < 50% received chemo-immunotherapy. In non-squamous NSCLC, treatment consisted mainly of carboplatin, pemetrexed, and pembrolizumab according to KEYNOTE-189 [20], or nivolumab plus ipilimumab with two cycles of carboplatin and pemetrexed according to CheckMate 9LA [21]. In squamous NSCLC, pemetrexed was replaced by paclitaxel or nab-paclitaxel; treatment options included pembrolizumab-based chemo-immunotherapy, nivolumab plus ipilimumab with platinum-doublet chemotherapy, or cemiplimab combined with platinum-based chemotherapy. Maintenance therapy consisted of immunotherapy alone. Patients with PD-L1 expression ≥ 50% received single-agent immunotherapy with pembrolizumab (KEYNOTE-024) [22], atezolizumab (IMpower110) [23], or cemiplimab (EMPOWER-Lung 1) [24].

### CT examination

Baseline contrast-enhanced CT examinations were performed on 128-slice multidetector CT scanners at the two participating institutions: a Philips Incisive scanner at Center 1 and a GE Optima scanner at Center 2.

At Center 1, CT acquisitions were performed using 100 kVp, a pitch of 1.20, and automatic tube-current modulation (DoseRight Index 18; tube-current range, 80–200 mA). A detector collimation of 64 × 0.625 mm was used, and images were reconstructed at 1-mm intervals.

At Center 2, CT acquisitions were performed using 120 kVp, 400 mA, a pitch of 0.96, and a total collimation width of 64 mm. Images were acquired with a slice thickness of 1–3 mm and reconstructed at 1-mm intervals.

In both centers, whole-body multiphasic CT examinations were acquired as part of routine oncologic staging after intravenous administration of iodinated contrast medium (350 mgI/mL) at a flow rate of 3.0–3.5 mL/s, with the iodine dose adjusted to lean body weight. Arterial, portal venous, and delayed phases were obtained using automated bolus tracking.

### Assessment of treatment response

Therapeutic response was assessed using contrast-enhanced chest CT at baseline and at the first follow-up after treatment initiation according to RECIST version 1.1 and iRECIST guidelines, which account for the distinctive response patterns observed in patients receiving immunotherapy [25–27].

Tumor response was categorized as follows: Complete response (CR): Complete disappearance of all target lesions; Partial response (PR): A minimum of 30% reduction in the sum of the longest diameters of target lesions compared to baseline measurements; Stable disease (SD): Insufficient shrinkage to meet the criteria for PR and insufficient increase to meet the criteria for PD; Progressive disease (PD): A minimum of 20% increase in the sum of the longest diameters of target lesions, or the emergence of new lesions. Nonetheless, according to iRECIST, progressive disease was confirmed by short-term follow-up imaging (within 4–8 weeks) to ensure validity.

Using these criteria, patients were divided into two outcome categories:

- Responders (R): Patients who achieved CR, PR, or SD collectively referred to as the disease control rate (DCR).
- Non-responders (NR): Patients exhibiting PD.

### Radiomic analysis

**Fig. 2:**
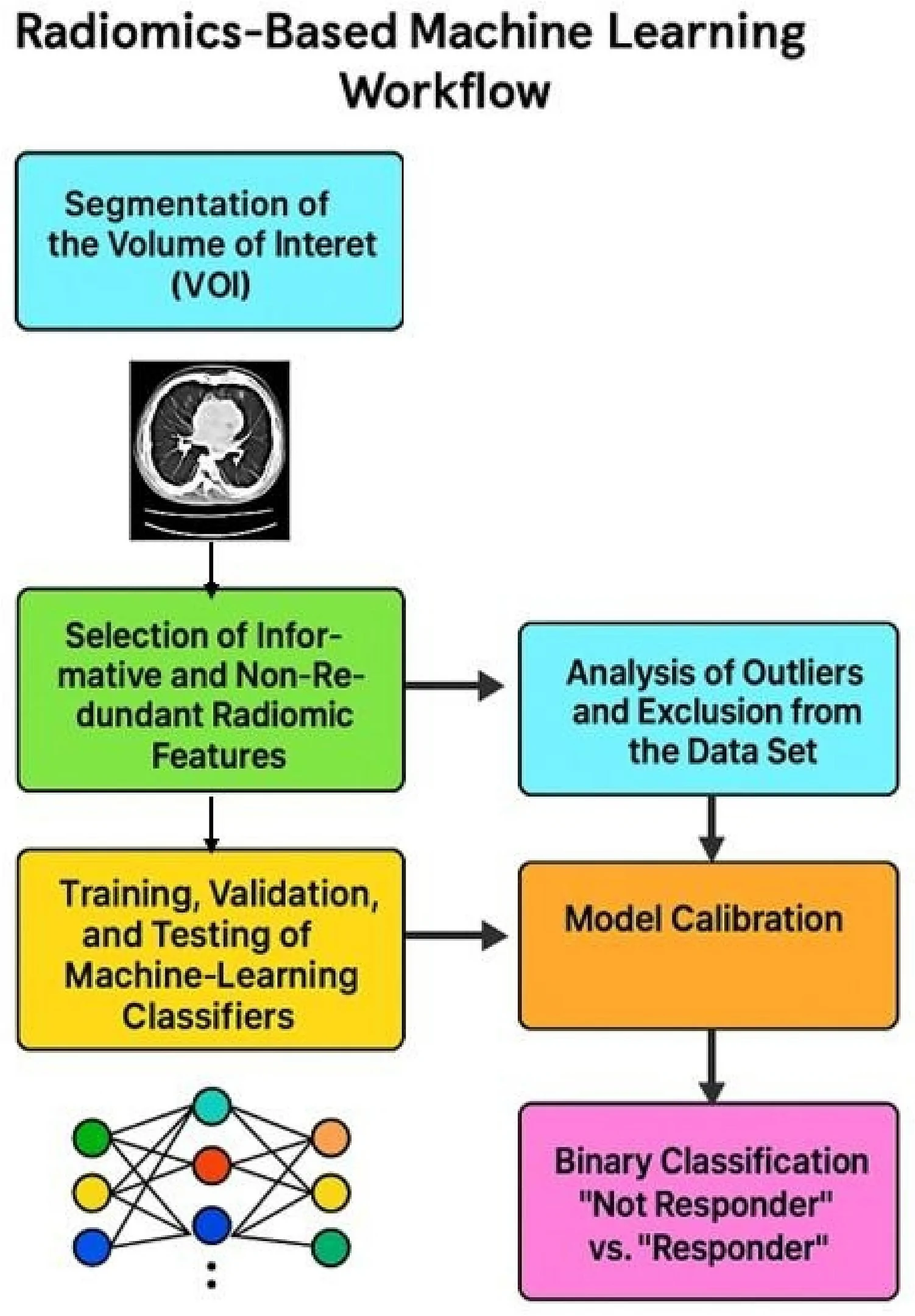
Radiomic-based machine learning workflow of the study

Radiomic analysis was performed in accordance with the image biomarker standardization initiative (IBSI) guidelines using the Trace4Research™ platform (v2.1.03, Deep Trace Technologies), ensuring a standardized and automated workflow (Fig. 2).

**Fig. 3:**
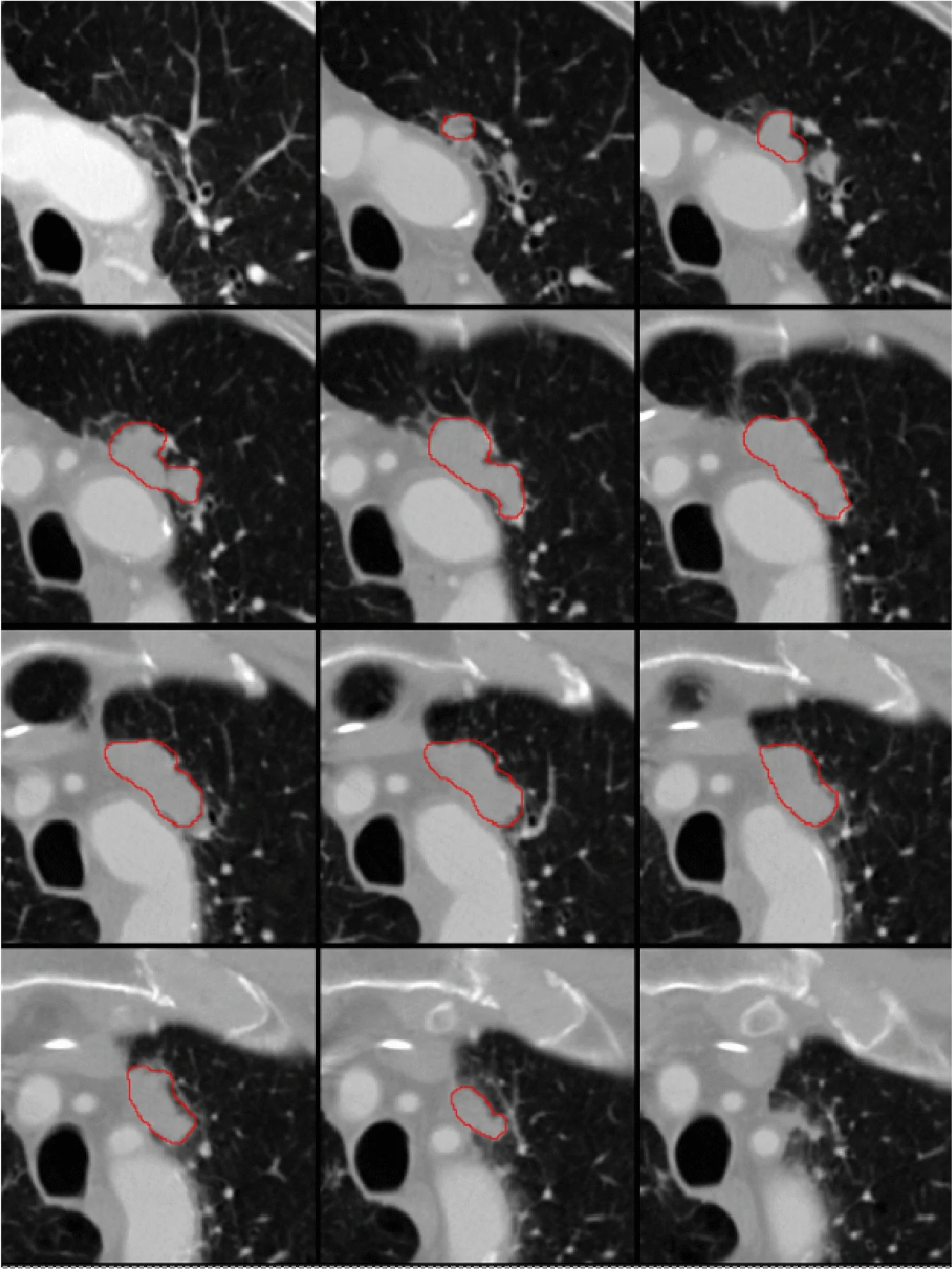
Example of a thoracic CT slice displayed in the *Deep Trace* software interface. The image shows a lung lesion undergoing manual segmentation, with the tumor margins delineated in red. The lesion appears as a soft-tissue–density mass with irregular contours. The software provides quantitative radiomic measurements, including an estimated lesion area of approximately 3 × 10^3^ mm^2^ and a volume of 2 × 10^5^ m

- Segmentation and preprocessing: Tumor volumes of interest (VOIs) were manually segmented slice by slice by one radiologist with eight years of experience in thoracic imaging, blinded to clinical and histopathological data. Segmentation quality and anatomical plausibility were subsequently reviewed by a second radiologist with six years of experience, and any necessary refinements were made by consensus. For each patient, a single volume of interest corresponding to the primary lung tumor was segmented. Metastatic lesions were not included in the radiomic analysis. Tumor volumes of interest were manually segmented on axial portal venous-phase CT images. Although multiphasic acquisitions were available, the portal venous phase was selected for all patients because it was consistently available across both centers and provided a uniform contrast-enhanced dataset for lesion delineation and radiomic feature extraction, thereby reducing phase-related variability. During the segmentation process, the radiologist was allowed to modify window settings as needed at their discretion. Images were subsequently resampled to isotropic 1-mm voxels using linear interpolation before feature extraction (Fig. 3).
- Feature harmonization: To reduce inter-scanner variability, radiomic features were harmonized using a ComBat-based approach implemented within the Trace4Research™ software, with scanner-related effects modeled as batch variability. Harmonization was performed independently of outcome labels.
- Feature extraction: IBSI-compliant radiomic features were extracted from original and filtered images (wavelet, square, square root, logarithmic, exponential, gradient, and Laplacian of Gaussian). Features included shape descriptors, first-order intensity and histogram features, and texture features derived from gray-level co-occurrence (GLCM), run-length (GLRLM), size-zone (GLSZM), neighboring gray-tone difference (NGTDM), and gray-level dependence (NGLDM) matrices. Intensity discretization was performed using a fixed bin width of 25. Additionally, 2048 deep features were extracted from 224 × 224 × 71 voxel images using a pretrained ResNet50 network.
- Feature selection and outlier detection: To assess robustness to segmentation variability, feature stability was evaluated using automated perturbations of the manual VOIs and image-rotation procedures implemented in the Trace4Research™ platform. Features with an intraclass correlation coefficient [ICC (2,1)] < 0.75 were considered insufficiently stable and were excluded. Features with low variance (coefficient of variation < 0.1) or low mutual information (< 0.3) were also excluded. A genetic algorithm optimized a custom fitness function aimed at maximizing feature relevance and minimizing redundancy, quantified using symmetric uncertainty. Outliers were identified using the Mahalanobis distance (> 3 standard deviations) and excluded.
- Machine learning and validation: Five machine learning classifiers—random forest, support vector machine, K-nearest neighbors, multilayer perceptron, and logistic regression—were trained for the binary classification task (“Responder” vs. “Not Responder”), using treatment response as the reference standard. Class imbalance was addressed through oversampling using the adaptive synthetic sampling method (ADASYN), and dimensionality reduction was performed using principal component analysis (PCA) and Fisher discriminant ratio (FDR). Models were developed separately in chemo-immunotherapy and immunotherapy cohorts using nested random tenfold cross-validation. To reduce the risk of information leakage, cross-validation splitting was performed before oversampling, feature normalization, principal component analysis, model training, and hyperparameter optimization. Oversampling was applied exclusively within the training partitions. All VOIs from the same patient were assigned to the same fold. For each algorithm, three ensembles of 100 classifiers were trained.

The external validation cohort from Center 2 was kept fully independent throughout model development and was used only for final external validation. It was not used for feature selection, classifier selection, hyperparameter optimization, threshold selection, or oversampling.

Performance, Explainability, and Calibration: Model performance was evaluated using ROC-AUC, accuracy, sensitivity, specificity, positive predictive value (PPV), negative predictive value (NPV), and F1-score, considering the “Not Responder” class as positive. Threshold-dependent performance metrics were primarily calculated using a prespecified predicted-probability cutoff of 0.50. Additional software-generated ROC-derived operating points, corresponding to more balanced sensitivity and specificity, were reported for descriptive purposes only and were not used for model selection or for the primary performance assessment. The model with the highest mean ROC-AUC on the internal test set was selected, and external validation was used to assess robustness and generalizability. Model explainability was addressed through ensemble-based feature analysis, identifying and ranking the most informative radiomic features using nonparametric statistical testing with correction for multiple comparisons. Detailed information on the selected predictors in the chemo-immunotherapy cohort and on the top 10 ranked predictors in the immunotherapy cohort is provided in Supplementary Tables S1 and S2, respectively.

Model calibration was assessed using reliability diagrams with 10 equally spaced bins, along with expected calibration error (ECE) and Brier score (BS).

### Statistical analysis

Statistical analysis was performed in two sequential phases.

#### Clinical data analysis

Associations between clinical variables and treatment response were evaluated using logistic regression analyses performed with Stata software (version 17.0; StataCorp LLC, College Station, TX, USA). Univariate logistic regressions were initially conducted to identify candidate predictors, excluding tumor type and therapeutic regimen, which were used as grouping variables. Variables with *p* < 0.10 in univariate analysis were included in the multivariate logistic regression model. Statistical significance in the multivariate analysis was set at *p* < 0.05.

#### Radiomic data analysis

The relationship between radiomic features and treatment response was assessed using the embedded statistical tools of the Trace4Research platform. Radiomic feature distributions in responder and non-responder groups were summarized using medians and 95% confidence intervals and visualized graphically. Group comparisons were performed using the nonparametric Wilcoxon rank-sum test (Mann–Whitney U test).

*P*-values were adjusted for multiple comparisons using the Bonferroni–Holm correction. For all *p*-values reported in this manuscript, statistical significance was set at 0.05 (*) and 0.005 (**).

Both models underwent internal and external validation using 1000 bootstrap resamples to assess discrimination and estimate 95% confidence intervals for the area under the receiver operating characteristic curve.

## Results

### Clinical variables and response to therapy

Patients’ characteristics and main clinical variables of the complete dataset are reported in Table 1 The median tumor volume of the first cohort and second cohort was comparable (median 39 cc, range2–507 cc vs. median 39 cc, range1–758 cc). In univariate regression analysis, the only predictor variable that was excluded was the occurrence of previous toxicity episodes after chemotherapy.

**Table 1:** Baseline characteristics of the study population. This table summarizes the demographic and clinical characteristics of the 130 patients included in the study

Variable	*n*	%
*Sex*		
• Male	77	59.2
• Female	53	40.8
Average Age	66.8 (37–85)	
*Smoke*		
• Yes	76	58.5
• No	54	41.5
*Stage*		
• III	37	28.5
• IV	93	71.5
PD-L1		
• < 50%	68	52.3
• > 50%	62	47.7
*Histology*		
• Adenocarcinoma	81	62.3
• Squamous Cell Carcinoma	38	29.2
• Large Cell	2	1.5
• Undifferentiated	9	6.9
*Toxicity*		
• Yes	43	33.1
• No	87	66.9
*Comorbidity*		
• Yes	51	39.2
• No	79	60.8

At multivariable logistic regression analysis, active smoking was independently associated with treatment response (OR 1.76, 95% CI 1.27–9.37, *p* = 0.015). Conversely, elevated LDH (OR 0.11, 95% CI 0.07–0.58, *p* = 0.003) and high CEA levels (OR 0.15, 95% CI 0.09–0.81, *p* = 0.019) were significantly associated with lack of response. Adenocarcinoma histology, comorbidities, and PD-L1 expression did not reach statistical significance (see Figure S1).

### Radiomic-based machine learning modeling of therapy response

Datasets from Groups A and B were classified according to therapy response (responder vs. non-responder) and used to train, validate, and test five supervised ML models (random forest, SVM, KNN, MLP, logistic regression) via nested tenfold cross-validation. A total of 3738 IBSI-compliant radiomic and deep learning features were extracted from segmented VOIs. In the development cohort, the chemo-immunotherapy group included 37 responders and 15 non-responders, whereas the immunotherapy group included 22 responders and 16 non-responders. In the external validation cohort, each treatment group included 10 responders and 10 non-responders.

**Fig. 4:**
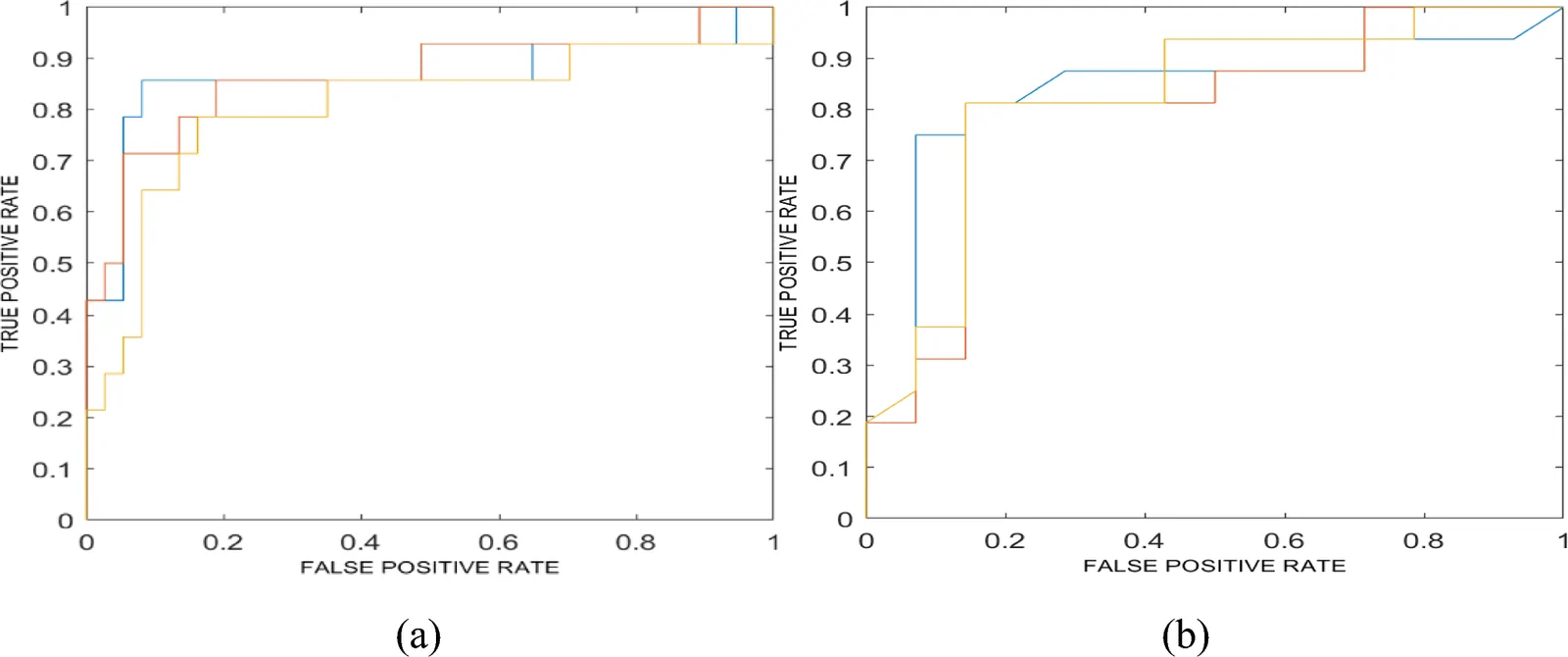
**a-b** ROC curves for each ensemble of the SVM models from internal testing with aggregated predictions, respectively, for Group A and B

**Fig. 5:**
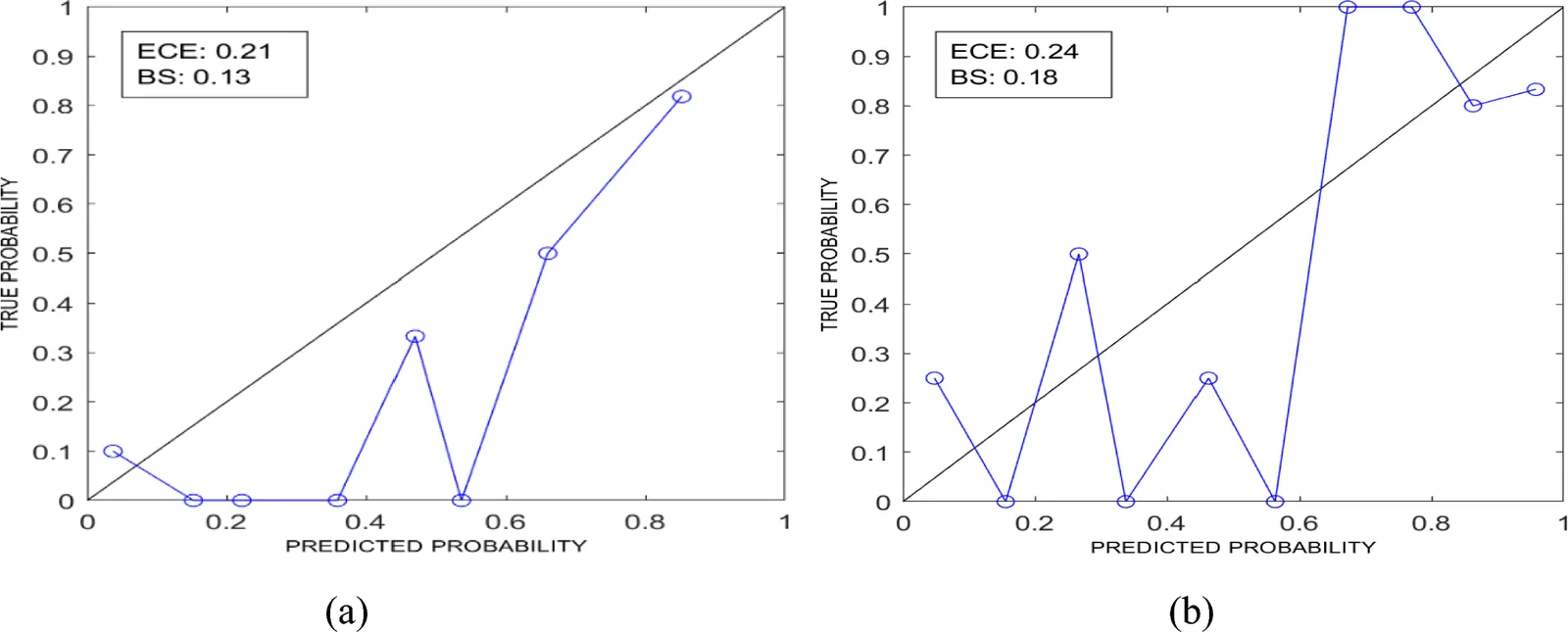
**a-b** Reliability diagrams for SVM model, from internal testing with aggregated predictions, respectively, for Group A and B. Predicted probability (consensus) is compared with true probability of being in the positive class. ECE and BS are reported

**Fig. 6:**
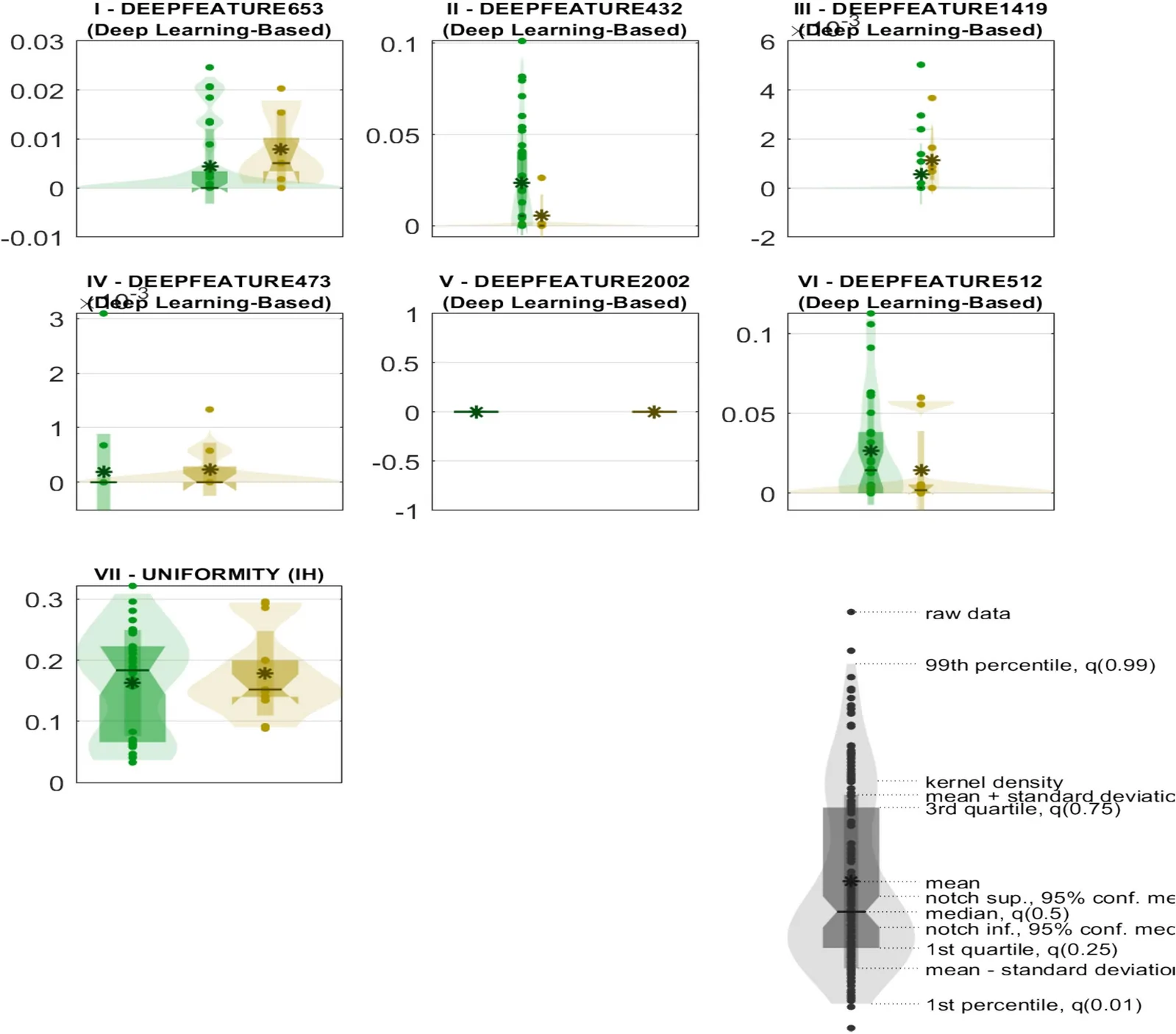
Distribution of the selected radiomic predictors in the chemo-immunotherapy cohort. Violin and box plots show the distribution of the selected features in responders and non-responders. Exact feature nomenclature, feature family, median values, and adjusted p-values are reported in Supplementary Table S1

Group A (chemo-immunotherapy): 7 features were selected after outlier removal (1NR) for the analysis. Among the tested machine learning algorithms, the support vector machine achieved the best performance in the chemo-immunotherapy cohort, with a ROC-AUC of 0.90 [0.82–0.97], accuracy of 82% [76–88], sensitivity of 82% [68–95], specificity of 83% [76–89], PPV of 67% [55–79], NPV of 94% [90–98], and an F1-score of 68% [56–81] (*p* < 0.005). Performance metrics for all evaluated models are summarized in Table 2, while the ROC curve of the top-performing model is shown in Fig. 4a. Model calibration and reliability are illustrated by the reliability diagram in Fig. 5a. The distribution of the radiomic predictors across responder and non-responder classes is shown using violin and box plots in Fig. 6.

**Table 2:** Performance metrics for model tested for the different binary classification of interest in Group A

	Training (50% threshold)	Validation (50% threshold)	Internal Testing (50% threshold)	Internal Testing (40% threshold)
*Model of three ensembles of Random Forest classifiers*				
ROC-AUC (%) [95%]	100** [100–100]	90** [87–92]	89** [81–96]	86
Accuracy (%) [95%]	100** [100–100]	84** [82–86]	86** [81–92]	86
Sensitivity (%) [95%]	100** [100–100]	74** [70–79]	75** [61–89]	86
Specificity (%) [95%]	100** [100–100]	88** [85–90]	91** [86–96]	86
PPV (%) [95%]	100** [100–100]	78** [75–82]	79** [66–92]	71
NPV (%) [95%]	100** [100–100]	92** [91–93]	91** [87–96]	94
*Model of three ensembles of Support Vector Machine classifiers*				
ROC-AUC (%) [95%]	97** [96–97]	87** [84–89]	90** [82–97]	84
Accuracy (%) [95%]	95** [94–95]	81** [79–83]	82** [76–88]	78
Sensitivity (%) [95%]	87** [87–88]	80** [76–85]	82** [68–95]	79
Specificity (%) [95%]	97** [97–97]	81** [78–83]	83** [76–89]	78
PPV (%) [95%]	93** [92–93]	68** [65–72]	67** [55–79]	58
NPV (%) [95%]	95** [95–96]	94** [92–95]	94** [90–98]	91
*Model of three ensembles of K-Nearest Neighbors classifiers*				
ROC-AUC (%) [95%]	96** [96–96]	85** [82–87]	83** [75–91]	87
Accuracy (%) [95%]	90** [89–90]	78** [76–80]	79** [73–84]	78
Sensitivity (%) [95%]	74** [72–75]	40 [35–45]	32 [16–48]	79
Specificity (%) [95%]	96** [95–96]	92** [90–94]	96** [92–99]	78
PPV (%) [95%]	87** [86–89]	72** [66–78]	72** [48–96]	58
NPV (%) [95%]	91** [90–91]	82** [81–84]	80** [75–85]	91
*Model of three ensembles of Multilayer Perceptron classifiers*				
ROC-AUC (%) [95%]	92** [91–94]	85** [81–88]	79** [69–90]	80
Accuracy (%) [95%]	90** [88–92]	84** [82–86]	87** [82–91]	71
Sensitivity (%) [95%]	80** [79–82]	62** [56–67]	52 [34–69]	71
Specificity (%) [95%]	94** [92–95]	92** [90–94]	99** [97–100]	70
PPV (%) [95%]	90** [87–92]	83** [78–87]	95** [84–100]	48
NPV (%) [95%]	92** [91–93]	89** [87–90]	87** [82–91]	87
*Model of three ensembles of Logistic Regression classifiers*				
ROC-AUC (%) [95%]	87** [86–88]	81** [77–85]	84** [72–96]	82
Accuracy (%) [95%]	90** [90–91]	84** [82–86]	85** [79–91]	78
Sensitivity (%) [95%]	68** [66–70]	52 [47–58]	58 [41–76]	79
Specificity (%) [95%]	99** [98–99]	96** [94–97]	95** [91–99]	78
PPV (%) [95%]	96** [95–96]	86** [82–90]	84** [69–99]	58
NPV (%) [95%]	89** [89–90]	87** [85–88]	88** [82–93]	91

The primary classification threshold was 0.50. Additional software-generated ROC-derived thresholds were reported for descriptive purposes only and were not used for model selection

Bold values indicate the performance metrics of the selected Support Vector Machine (SVM) model

**Fig. 7:**
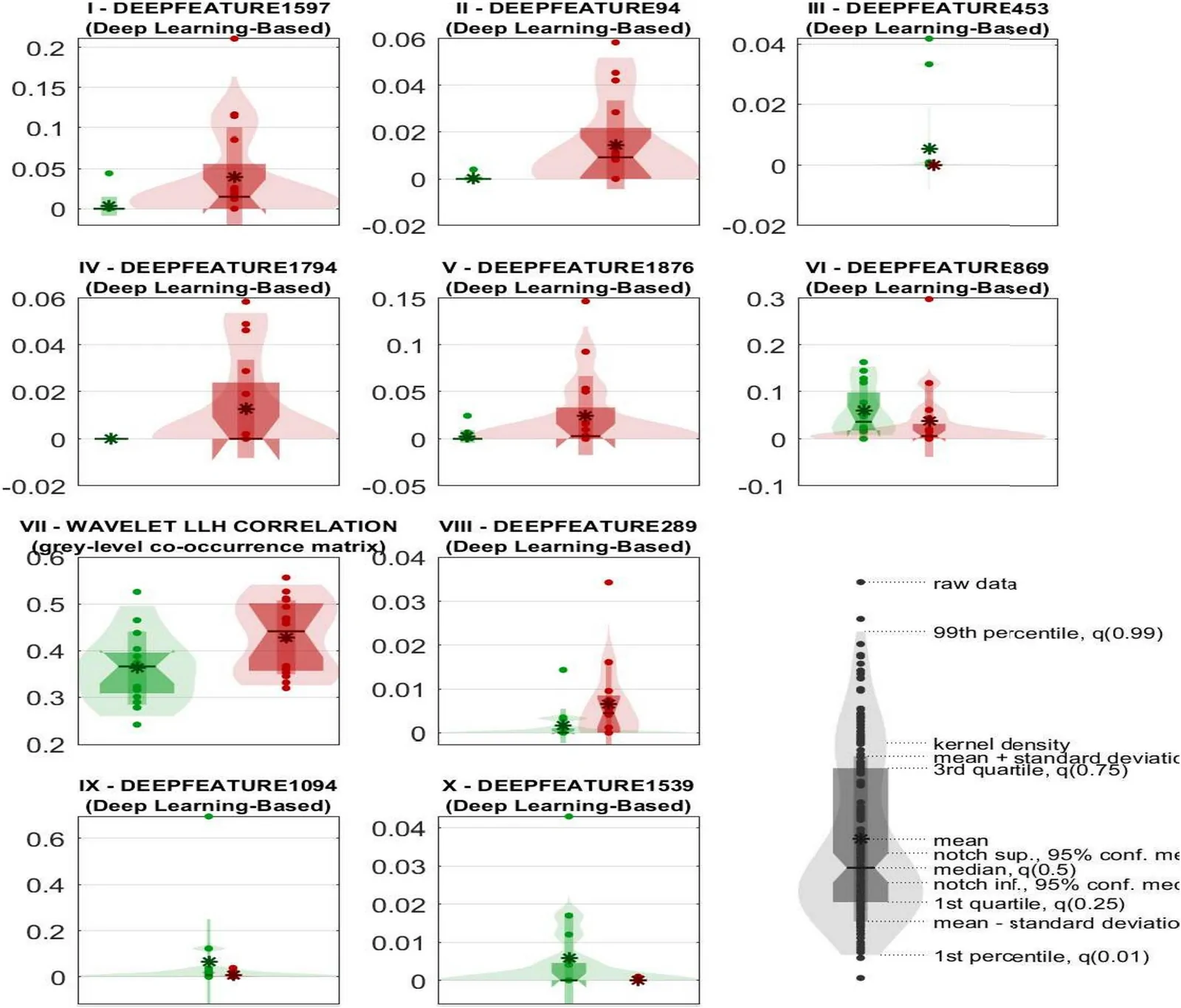
Distribution and ranking of the top selected radiomic and deep learning predictors in the immunotherapy cohort. Violin and box plots illustrate feature distributions in responders, defined as patients achieving disease control, and non-responders, defined as patients with progressive disease. The displayed predictors represent the highest-ranked features identified by the ensemble-based analysis. Exact feature nomenclature, feature family, and statistical results are provided in Supplementary Table S2

Group B (immunotherapy): From segmented VOIs, a total of 29 radiomic features were retained after feature selection. The support vector machine again performed best: ROC-AUC 84% [73–95], accuracy 77% [68–86], sensitivity 82% [69–94], specificity 70% [56–84], PPV 80% [71–90], NPV 83% [72–94] (*p < 0.05, **p < 0.005). Performance metrics are reported in Table 3, whereas the ROC curve is shown in Fig. 4b. The reliability diagram is shown in Fig. 5b, whereas Fig. 7 presents violin and box plots of the selected radiomic predictors.

**Table 3:** Performance metrics for each model tested for the different binary classification of interest in Group B

	Training (50% threshold)	Validation (50% threshold)	Internal Testing (50% threshold)	Internal Testing (40% threshold)
*Model of three ensembles of Random Forest classifiers*				
ROC-AUC (%) [95%]	100** [100–100]	82** [78–86]	78** [67–90]	74
Accuracy (%) [95%]	100** [100–100]	69** [66–72]	67** [58–76]	70
Sensitivity (%) [95%]	100** [100–100]	71** [66–76]	72** [57–86]	56
Specificity (%) [95%]	100** [100–100]	67** [62–72]	63* [49–78]	86
PPV (%) [95%]	100** [100–100]	75** [72–79]	72** [62–83]	82
NPV (%) [95%]	100** [100–100]	73** [69–77]	73** [60–86]	63
*Model of three ensembles of Support Vector Machine classifiers*				
ROC-AUC (%) [95%]	100** [100–100]	83** [80–86]	84** [73–95]	80
Accuracy (%) [95%]	97** [96–98]	69** [67–72]	77** [68–86]	83
Sensitivity (%) [95%]	100** [100–100]	94** [92–97]	82** [69–94]	81
Specificity (%) [95%]	94** [92–96]	41 [36–46]	70* [56–84]	86
PPV (%) [95%]	96** [95–97]	69** [67–72]	80** [71–90]	87
NPV (%) [95%]	100** [100–100]	89** [85–94]	83** [72–94]	80
*Model of three ensembles of K-Nearest Neighbors classifiers*				
ROC-AUC (%) [95%]	92** [91–93]	88** [85–90]	78** [66–91]	76
Accuracy (%) [95%]	77** [75–78]	74** [71–77]	79** [70–87]	67
Sensitivity (%) [95%]	74** [70–77]	66** [62–71]	72** [57–86]	50
Specificity (%) [95%]	80** [77–83]	83** [79–87]	85** [74–96]	86
PPV (%) [95%]	87** [85–89]	85** [82–89]	89** [81–97]	80
NPV (%) [95%]	80** [78–82]	76** [72–79]	80** [70–89]	60
*Model of three ensembles of Multilayer Perceptron classifiers*				
ROC-AUC (%) [95%]	99** [99–100]	91** [88–93]	83** [73–92]	73
Accuracy (%) [95%]	86** [84–87]	58** [56–61]	56* [49–62]	53
Sensitivity (%) [95%]	73** [70–77]	28 [24–33]	18 [6–31]	13
Specificity (%) [95%]	100** [99–100]	92** [90–95]	97** [90–100]	100
PPV (%) [95%]	100** [99–100]	81** [75–88]	94* [79–100]	100
NPV (%) [95%]	81** [79–83]	58** [55–60]	53** [45–60]	50
*Model of three ensembles of Logistic Regression classifiers*				
ROC-AUC (%) [95%]	100** [100–100]	88** [85–91]	82** [69–94]	79
Accuracy (%) [95%]	100** [100–100]	76** [73–78]	80** [71–88]	80
Sensitivity (%) [95%]	100** [100–100]	74** [69–78]	78** [65–92]	75
Specificity (%) [95%]	100** [100–100]	78** [73–82]	80** [66–94]	86
PPV (%) [95%]	100** [100–100]	84** [81–87]	87** [79–96]	86
NPV (%) [95%]	100** [100–100]	80** [76–83]	82** [71–93]	75

The primary classification threshold was 0.50. Additional software-generated ROC-derived thresholds are reported for descriptive purposes only and were not used for model selection.Bold values indicate the performance metrics of the selected support vector machine (SVM) model. Bold values indicate the performance metrics of the selected support vector machine (SVM) model

An external testing set of 40 samples (20 patients for each group evenly distributed between two classes) was used to validate the models. During external validation, the models showed moderate discriminative performance, with ROC-AUCs of 0.78 for Group A and 0.70 for Group B.

Bootstrap analysis confirmed stable internal discriminative performance for both models. As shown in Figure S2 in the Supplementary Material, Model 1 (Group A) consistently achieved higher AUC values than Model 2 (Group B), with an expected decrease in discriminative performance observed during external validation.

Overall probabilistic performance was evaluated using the Brier score. As shown in Figure S3, Model 1 demonstrated lower prediction error than Model 2 and maintained superior probabilistic accuracy in the external cohort.

## Discussion

In this study, CT-based radiomics machine learning models were developed to predict treatment response in two cohorts of patients with advanced-stage NSCLC receiving chemo-immunotherapy or immunotherapy alone. In line with the clinical objective of early treatment evaluation, response assessment focused on disease control rather than tumor shrinkage alone. Stable disease was therefore included among responders, reflecting a commonly adopted endpoint in advanced NSCLC, particularly in immunotherapy-based settings. Nevertheless, stable disease may represent a biologically heterogeneous condition and does not necessarily correspond to objective tumor response. Accordingly, the present findings should be interpreted primarily in terms of early disease control versus progression, rather than objective response alone.

Among the evaluated supervised machine learning models, the support vector machine (SVM) consistently achieved the highest predictive performance in both cohorts, confirming its robustness and suitability for high-dimensional radiomic data in relatively small samples.

In Group A, the best-performing model showed balanced sensitivity (82%) and specificity (83%), with a particularly high negative predictive value (94%), indicating strong reliability in identifying patients unlikely to benefit from therapy and potentially supporting earlier treatment reassessment. The more moderate positive predictive value (67%) suggests that further improvements could be achieved by integrating complementary information such as genomics, radiogenomics, or molecular biomarkers.

In Group B, model performance remained high (ROC-AUC = 0.84, sensitivity 82%, specificity 70%), confirming the reproducibility of the SVM classifier in a different therapeutic context. Calibration and reliability metrics further supported the stability of the proposed models.

These findings are consistent with previous studies demonstrating the value of CT-based radiomics in predicting treatment response and survival in lung cancer. Bao et al. reported an AUC of 0.84 for predicting response to chemo-immunotherapy using CT-based radiomics [28]. Wang et al. achieved AUCs of 0.70 in the training cohort and 0.60 in the validation cohort [29], while delta-radiomic models showed improved performance with AUCs ranging from 0.81 to 0.86 [30]. Liu et al. further demonstrated reliable prediction of immunotherapy response in patients with high PD-L1 expression (AUC = 0.859), with accuracy comparable to that observed in our cohort [31].

During external validation, the models showed moderate discriminative performance, with ROC-AUCs of 0.78 for Group A and 0.70 for Group B.

The predictive features identified in the two cohorts were largely non-overlapping. This may suggest different imaging patterns across treatment settings; however, it should not be interpreted as direct evidence of treatment-specific biological signatures. The selected features were mainly related to intensity distribution and texture homogeneity in the chemo-immunotherapy cohort, and to higher-order spatial complexity and heterogeneity in the immunotherapy cohort. Any biological interpretation remains hypothesis-generating and requires validation against histopathological, molecular, or immune-microenvironmental markers.

Comparison between the two SVM ensemble models revealed differences in classification behavior. The chemo-immunotherapy model showed more balanced discrimination across thresholds, whereas the immunotherapy model favored sensitivity at the expense of specificity, suggesting potential use in clinical scenarios prioritizing sensitivity.

Multivariate analysis identified smoking status and the tumor markers LDH and CEA as independent predictors of treatment response. Active smoking was associated with a higher likelihood of response, consistent with prior evidence indicating improved outcomes with immune checkpoint inhibitors in ever-smokers [32]. In contrast, elevated LDH and CEA levels were associated with reduced response probability, reflecting more aggressive disease biology, in agreement with previous reports linking these biomarkers to worse outcomes [33, 34]. Notably, none of the identified clinical predictors showed significant associations with the selected radiomic features, suggesting that radiomics may capture information not fully represented by the investigated conventional clinical variables. However, the potential incremental value of integrating clinical and radiomic information should be formally assessed in larger multimodal predictive models. [35].

A key methodological distinction of the present study lies in the adoption of an ensemble learning framework integrating multiple classifiers, which contributed to improved robustness compared with single-model approaches.

The proposed models may have relevant clinical implications by enabling early identification of patients unlikely to benefit from therapy, potentially optimizing treatment selection without additional cost or invasive procedures.

Several limitations should be acknowledged, including the retrospective design, the relatively small sample size, and the limited interpretability of deep learning features. Although an independent external cohort was included, each treatment-specific validation subgroup comprised only 20 patients and was balanced by outcome class. Therefore, the external performance estimates should be interpreted cautiously, and prevalence-dependent metrics such as PPV and NPV may not be directly transferable to real-world clinical populations. Given the limited sample size relative to the initial high-dimensional radiomic and deep-feature space, the risk of overfitting and instability in feature selection cannot be completely excluded.

Although cross-validation, feature selection, and ComBat-based harmonization were applied to mitigate overfitting and scanner-related variability, residual heterogeneity cannot be fully excluded [36–38]. In addition, a formal interobserver reproducibility analysis based on independent segmentations by multiple readers was not performed. Although ICC-based feature-stability filtering using automated segmentation perturbations was applied, this approach does not replace a dedicated interobserver reproducibility assessment.

End-to-end voxel-based deep learning approaches were not adopted due to sample size constraints and overfitting risk in a multicenter setting. Instead, a hybrid strategy based on deep-feature extraction from a pretrained ResNet50 network was used to balance representational power and robustness.

Future studies will focus on expanding the cohort through additional centers to enable large-scale validation and on exploring end-to-end deep learning models in larger, more homogeneous datasets.

## Conclusions

In conclusion, radiomics-based machine learning models may represent a promising noninvasive tool for the early prediction of treatment response in patients with advanced NSCLC treated with chemo-immunotherapy or immunotherapy alone. Although the present study was based on a relatively small cohort, the models showed encouraging performance in both internal and external validation. Further multicenter studies are needed to confirm these findings and to clarify the added value of radiomics within integrated clinical decision-making frameworks.

## Supplementary Information

Below is the link to the electronic supplementary material.

Supplementary file1 (DOCX 230 KB)
