# Supplementary material for: CT radiomics machine learning for predicting treatment response in unresectable stage III-IV NSCLC: insights from dual chemo-immunotherapy and immunotherapy cohorts
Source: Radiol Med. 2026 Aug 10;131(9):1586–600. doi: 10.1007/s11547-026-02270-5 (PMC13658530; doi:10.1007/s11547-026-02270-5)
Supplement: Supplementary file 1 [file 11547_2026_2270_MOESM1_ESM.docx]

**Supplementary Materials**

**Figure S1** Multivariable logistic regression analysis for treatment response

**Figure S2** Discriminative performance of Model 1 and Model 2 assessed by the area under the receiver operating characteristic curve (AUC) using bootstrap validation (1,000 resamples). Model 1 demonstrated higher discriminative performance than Model 2, with a reduction in AUC observed for both models in the external validation cohort

**Figure S3** Probabilistic performance of Model 1 and Model 2 assessed using the Brier score based on bootstrap internal validation and external validation (1,000 resamples). Box plots show the median, interquartile range, and minimum–maximum values of the Brier score distributions, with lower values indicating better probabilistic accuracy. Model 1 consistently achieved lower Brier scores than Model 2, both in internal and external validation

**Supplementary Table S1**. Selected predictors in the chemo-immunotherapy cohort. Median values (95% CI) and uncorrected and corrected p-values are reported

| **#** | **Feature family** | **Feature nomenclature** | **Median in the negative class [95% CI]** | **Median in the positive class [95% CI]** | **Uncorrected p-value** | **Corrected p-value** |
| --- | --- | --- | --- | --- | --- | --- |
| **I** | **Deep Learning-Based** | DeepFeature653 | 8.40e-04 [-4.48e-03 - 6.16e-03] | 3.78e-02 [1.73e-02 - 5.82e-02] | < 0.005 | < 0.05 |
| **II** | **Deep Learning-Based** | DeepFeature432 | 5.38e-03 [-5.32e-03 - 1.61e-02] | 1.33e-01 [1.76e-02 - 2.49e-01] | < 0.005 | < 0.05 |
| **III** | **Deep Learning-Based** | DeepFeature1419 | 0.00e+00 [-2.34e-03 - 2.34e-03] | 3.54e-02 [-1.29e-02 - 8.37e-02] | < 0.05 | < 0.05 |
| **IV** | **Deep Learning-Based** | DeepFeature473 | 6.77e-04 [-1.73e-03 - 3.08e-03] | 9.57e-04 [-5.46e-02 - 5.65e-02] | 0.26 | 1.00 |
| **V** | **Deep Learning-Based** | DeepFeature2002 | 0.00e+00 [-1.76e-03 - 1.76e-03] | 1.51e-03 [-1.65e-02 - 1.96e-02] | 0.32 | 1.00 |
| **VI** | **Deep Learning-Based** | DeepFeature512 | 2.08e-02 [-6.62e-03 - 4.82e-02] | 3.06e-02 [-8.64e-02 - 1.48e-01] | 0.59 | 1.00 |
| **VII** | **Intensity Histogram** | Uniformity | 1.83e-01 [1.42e-01 - 2.25e-01] | 1.52e-01 [1.26e-01 - 1.78e-01] | 0.81 | 1.00 |

**Supplementary Table S2.** Top 10 predictors in the immunotherapy cohort. Median values (95% CI) and uncorrected and corrected p-values are reported.

| **#** | **Feature family** | **Feature nomenclature** | **Median in the positive class (95% CI)** | **Median in the negative class (95% CI)** | **Uncorrected p-value** | **Corrected p-value** |
| --- | --- | --- | --- | --- | --- | --- |
| **I** | Deep Learning-Based | DeepFeature1597 | 1.49e-02 (-7.05e-03 - 3.68e-02) | 0.00e+00 (0.00e+00 - 0.00e+00) | < 0.005 | 0.14 |
| **II** | Deep Learning-Based | DeepFeature94 | 9.30e-03 (8.03e-04 - 1.78e-02) | 0.00e+00 (0.00e+00 - 0.00e+00) | < 0.05 | 0.30 |
| **III** | Deep Learning-Based | DeepFeature453 | 0.00e+00 (0.00e+00 - 0.00e+00) | 0.00e+00 (-2.35e-04 - 2.35e-04) | < 0.05 | 0.46 |
| **IV** | Deep Learning-Based | DeepFeature1794 | 0.00e+00 (-9.35e-03 - 9.35e-03) | 0.00e+00 (0.00e+00 - 0.00e+00) | < 0.05 | 0.54 |
| **V** | Deep Learning-Based | DeepFeature1876 | 2.91e-03 (-1.03e-02 - 1.61e-02) | 0.00e+00 (0.00e+00 - 0.00e+00) | < 0.05 | 0.92 |
| **VI** | Deep Learning-Based | DeepFeature869 | 6.03e-03 (-5.99e-03 - 1.81e-02) | 3.65e-02 (-6.18e-03 - 7.92e-02) | < 0.05 | 1.00 |
| **VII** | Gray-Level Co-Occurrence Matrix | Wavelet LLH Correlation | 4.42e-01 (3.85e-01 - 4.99e-01) | 3.66e-01 (3.23e-01 - 4.09e-01) | 0.05 | 1.00 |
| **VIII** | Deep Learning-Based | DeepFeature289 | 4.49e-03 (1.15e-03 - 7.83e-03) | 0.00e+00 (-5.38e-04 - 5.38e-04) | 0.07 | 1.00 |
| **IX** | Deep Learning-Based | DeepFeature1094 | 0.00e+00 (-3.17e-03 - 3.17e-03) | 8.30e-03 (-2.90e-03 - 1.95e-02) | 0.09 | 1.00 |
| **X** | Deep Learning-Based | DeepFeature1539 | 0.00e+00 (0.00e+00 - 0.00e+00) | 0.00e+00 (-2.11e-03 - 2.11e-03) | 0.09 | 1.00 |
